# VIKOR Method for MAGDM Based on Q-Rung Interval-Valued Orthopair Fuzzy Information and Its Application to Supplier Selection of Medical Consumption Products

**DOI:** 10.3390/ijerph17020525

**Published:** 2020-01-14

**Authors:** Hui Gao, Linggang Ran, Guiwu Wei, Cun Wei, Jiang Wu

**Affiliations:** 1 School of Business, Sichuan Normal University, Chengdu 610101, China; gaohuisxy@sicnu.edu.cn; 2 School of Economics and Management, Chongqing University of Arts and Sciences, Chongqing 402160, China; 3 School of Statistics, Southwestern University of Finance and Economics, Chengdu 611130, China; weicun1990@163.com (C.W.); wujiang@swufe.edu.cn (J.W.)

**Keywords:** multiple attribute group decision making (MAGDM), q-rung interval-valued orthopair fuzzy sets (q-RIVOFSs), VIKOR method, q-RIVOF-VIKOR model, supplier selection, medical consumption products

## Abstract

The VIKOR model has been considered a viable tool for many decision-making applications in the past few years, given the advantages of considering the compromise between maximizing the utility of group and minimizing personal regrets. The q-rung interval-valued orthopair fuzzy set (q-RIVOFS) is a generalization of intuitionistic fuzzy set (IFS) and Pythagorean fuzzy set (PFS) and has emerged to solve more complex and uncertain decision making problems which IFS and PFS cannot handle. In this manuscript, the key innovation is to combine the traditional VIKOR model with q-RIVOFS to develop the q-rung interval-valued orthopair fuzzy VIKOR model. In the new developed model, to express more information, the attribute’s values in MAGDM problems are depicted by q-RIVOFNs. First of all, some basic theories and aggregation operators of q-RIVOFNs are simply introduced. Then we develop the origin VIKOR model to q-RIVOFS environment and briefly express the computing steps of this new established model. Thereafter, the effectiveness of the model is verified by an example of supplier selection of medical consumer products and through comparative analysis, the superiority of the new method is further illustrated.

## 1. Introduction

In view of the merits of the VIKOR model in considering the compromise between group utility maximization and individual regret minimization, in recent years, it has been recognized as a meaningful tool that can be applied to many decision areas. In previous literature, some traditional decision models have been applied to MADM problems, such as the ELECTRE model [1,2,3,4], the MABAC model [5,6,7], the COPRAS model [8,9], the TOPSIS model [10,11,12], The TODIM model [13,14,15], and the GRA model [16,17,18]. Compared with the above methods, the VIKOR model not only considers the objectivity of the decision maker and the complexity of the decision-making environment, but also considers the conflict criteria, so as to obtain more effective and accurate evaluation results. Du and Liu [19] developed the traditional VIKOR model into intuitionistic trapezoidal fuzzy environment. Park, et al. [20] established the IVIF-VIKOR model for MADM problems. Qin, et al. [21] came up with an extension of VIKOR model on the basis of interval type-2 fuzzy information. Ghadikolaei, et al. [22] extended the VIKOR model from the real number environment to the hesitating fuzzy linguistic environment, so it can better reflect the fuzziness of decision makers in making decisions in MADM problems. Wang, et al. [23] tried to expand the VIKOR model to the neutrosophic environment of triangular fuzzy, and applied it to evaluate the potential commercialization of emerging technologies. In order to select industrial robots more effectively, Narayanamoorthy, et al. [24] used an expanding VIKOR model on the foundation of interval intuitionistic hesitating fuzzy entropy. Later, some scholars Yang, et al. [25] determined the VIKOR model of language hesitation intuition to deal with the problem of MADM. Wang, et al. [26] established a VIKOR model based on projection in the context of picture fuzzy environment and used it in the risk assessment of construction projects. Wu, et al. [27] created the HFLTS-VIKOR model with possibility distributions.

Because of the uncertainty and decision problem of decision support system (DSS), in the practical DSS problem, we often cannot give the accurate evaluation value of the alternative to choose the best one. To overcome this problem, in 1965, the fuzzy set theory defined by Zadeh [28], initially applied membership functions instead of precise real numbers to describe the estimation results. Atanassov [29,30] added another metric that complements non-membership functions. In recent years, the proposed Pythagorean fuzzy set (PFS) [31,32] further expanded the scope of IFS, making the sum of squares of its membership degree and non-membership degree less than or equal to 1. Obviously, PFS is more extensive than IFS and can express more decision-making information, and the decision problems of IFS are special cases of PFS decision problems. In the previous literature, a great deal of research has been done on PFS. For example, Zhang and Xu [33] presented a combination of PFS and TOPSIS models to deal with MADM problems. In order to better understand the new fuzzy set of PFS, Peng and Yang [34] primarily put forward the division and subtraction operations of PFS. Reformat and Yager [35] applied Pythagorean fuzzy information to collaborative recommendation systems. Gou, et al. [36] studied some precious properties of continuous PFS. Garg [37] defined some new aggregation operators of PFS on the foundation of Einstein operations. Wu and Wei [38] came out some Hamacher aggregation operators of PFS to merge fuzzy information. Zeng, et al. [39] utilized the PFOWAWAD operator to study MADM issues under the context of PFS. Ren, et al. [40] established the PF-TODIM model. Combining with Pythagorean fuzzy environment, Wei and Lu [41] proposed a new MSM [42] operator. Wei [43] innovated some fuzzy interactive aggregation operators for arithmetic and geometric operations based on PFS. Wei and Lu [44] proposed some fuzzy power aggregation operators in the Pythagorean theorem. Wei and Wei [45] created ten cosine similarity measures in the fuzzy context of the Pythagorean theorem. Liang, et al. [46] studied some Bonferroni mean operators using Pythagorean fuzzy information. Liang, et al. [47] presented the PFGA operation based on Bonferroni mean aggregation operator. Combining the PFSs [31,32] and DHFSs [48,49], Wei and Lu [50] brought in the definition of the DHPFSs and proposed some DHPF-Hamacher aggregation operators. Peng, et al. [51] created some new PF information measures of MADM problems.

Nevertheless, to describe more decision information, Yager [52] later defined q-rung orthopair fuzzy sets (q-ROFSs), and based on PFS, the condition that the square sum of its membership and non-membership is less than or equal to 1 becomes that the sum of the qth power of the two is less than or equal to 1. Obviously, compared to IFS, q-ROFSs is more general, and PFS is a special case. Liu and Wang [53] put forward the q-ROFWA operator and the q-ROFWG operator. Wei, et al. [54] defined some q-rung orthopair fuzzy MSM operators including q-ROFMSM operator, q-ROFWMSM operator, q-ROFDMSM operator, q-ROFWDMSM operator. Wei, et al. [55] gave some q-ROF Heronian mean operators. Yang and Pang [56] provided some new definition of partitioned Bonferroni mean operators under q-ROFS. Wang, et al. [57] came up with some q-rung interval-valued fuzzy Hamy mean operators including q-RIVOFHM operator, q-RIVOFWHM operator, q-RIVOFDHM operator and q-RIVOFWDHM operator. Liu and Liu [58] offered some power Bonferroni mean operators with linguistic q-rung orthopair fuzzy information. Xu, et al. [59] gave the definition of q-RDHOFS and presented some q-RDHOF Heronian mean operators.

However, to date, it is clear that the VIKOR model with q-RIVOFNs information has not been studied. Therefore, it’s essential to take q-RIVOF-VIKOR model into consideration. The aim of our manuscript is to create an enlarged VIKOR model with the original VIKOR method and q-RIVOF information to settle MADM problems more effectively. Our manuscript is structured as: the definition, score function, accuracy function, operation rules, and some aggregation operators of q-RIVOFSs are briefly given in Section 2. The calculation process of traditional VIKOR model is briefly depicted in Section 3. Integrating the original VIKOR model with q-RIVOFNs information, the q-RIVOF-VIKOR technique is built and the calculation processes are simply shown in Section 4. An example of a vendor selection of healthcare consumer products has been illustrated by this new model and some comparisons between the q-RIVOF-VIKOR model and two q-RIVOFNs aggregation operators—including q-RIVOFWA and q-RIVOFWG operators—are also carried out to further explain merit of the new method in Section 5. Some conclusions of our manuscript are made in Section 6.

## 2. Preliminaries

Based on the theorems of q-ROFSs and the interval values, the essential definition and theorems of q-RIVOFSs are retrospected in brief below.

### 2.1. The q-RIVOFSs

**Definition** **1**  **[57].** *Let X be a fix set. A q-RIVOFSs has the following definition*:

(1) $$\tilde{P}=\left\{\langle x,\left({\tilde{\mu }}_{\tilde{P}}\left(x\right),{\tilde{\nu }}_{\tilde{P}}\left(x\right)\right)\rangle \left|x\in X\right.\right\}$$

*
where the function* ${\tilde{\mu }}_{\tilde{P}}\left(x\right)=\left[\mu_{\tilde{P}}^{L}\left(x\right),\mu_{\tilde{P}}^{U}\left(x\right)\right]:\;X\to \left[0,1\right]$ *defines the membership degree and the function *${\tilde{v}}_{\tilde{P}}\left(x\right)=\left[v_{\tilde{P}}^{L}\left(x\right),v_{\tilde{P}}^{U}\left(x\right)\right]:\;X\to \left[0,1\right]$ *defines the non-membership degree of the element* x∈X *to* $\tilde{P}$ *respectively, and, for every x ∈ X, it meets that* 

(2) $${\left(\mu_{\tilde{P}}^{U}\left(x\right)\right)}^{q}+{\left(v_{\tilde{P}}^{U}\left(x\right)\right)}^{q}\leq 1,q\geq 1.$$

${\tilde{\pi }}_{\tilde{P}}\left(x\right)=\left[\pi_{\tilde{P}}^{L}\left(x\right),\pi_{\tilde{P}}^{U}\left(x\right)\right]=\left[\sqrt[{q}]{1-\left({\left(\mu_{\tilde{P}}^{L}\left(x\right)\right)}^{q}+{\left(\nu_{\tilde{P}}^{L}\left(x\right)\right)}^{q}\right)},\sqrt[{q}]{1-\left({\left(\mu_{\tilde{P}}^{U}\left(x\right)\right)}^{q}+{\left(\nu_{\tilde{P}}^{U}\left(x\right)\right)}^{q}\right)}\right]$ *is the degree of indeterminacy membership. For convenience, we called* $\tilde{p}=\left(\left[\mu^{L},\mu^{U}\right],\left[\nu^{L},\nu^{U}\right]\right)$ *a q-RIVOFN*.

**Definition** **2** **[57].** *Let* $\tilde{p}=\left(\left[\mu^{L},\mu^{U}\right],\left[\nu^{L},\nu^{U}\right]\right)$ *be a q-RIVOFN, a score function S can be written as follows*:

(3) $$S\left(\tilde{p}\right)=\frac{1}{4}\left[\left(1+{\left(\mu^{L}\right)}^{q}-{\left(v^{L}\right)}^{q}\right)+\left(1+{\left(\mu^{U}\right)}^{q}-{\left(v^{U}\right)}^{q}\right)\right],\;S\left(\tilde{p}\right)\in \left[0,1\right].$$

**Definition** **3** **[57].** *Let* $\tilde{p}=\left(\left[\mu^{L},\mu^{U}\right],\left[\nu^{L},\nu^{U}\right]\right)$ *be a q-RIVOFN, an accuracy function* H *can be written as follows:*

(4) $$H\left(\tilde{p}\right)=\frac{{\left(\mu^{L}\right)}^{q}+{\left(\nu^{L}\right)}^{q}+{\left(\mu^{U}\right)}^{q}+{\left(\nu^{U}\right)}^{q}}{2},H\left(\tilde{p}\right)\in \left[0,1\right],$$

According to *S* and *H*, the order relation between two q-RIVOFNs will be obtained as below:

**Definition** **4** **[57].** *Let* ${\tilde{p}}_{1}=\left(\left[\mu_{1}^{L},\mu_{1}^{U}\right],\left[\nu_{1}^{L},\nu_{1}^{U}\right]\right)$* and *${\tilde{p}}_{2}=\left(\left[\mu_{2}^{L},\mu_{2}^{U}\right],\left[\nu_{2}^{L},\nu_{2}^{U}\right]\right)$ *be two q-RIVO FNs, assume that* $S\left({\tilde{p}}_{1}\right)=\frac{1}{4}\left[\left(1+{\left(\mu_{1}^{L}\right)}^{q}-{\left(v_{1}^{L}\right)}^{q}\right)+\left(1+{\left(\mu_{1}^{U}\right)}^{q}-{\left(v_{1}^{U}\right)}^{q}\right)\right]$ *and* $S\left({\tilde{p}}_{2}\right)=\frac{1}{4}\left[\left(1+{\left(\mu_{2}^{L}\right)}^{q}-{\left(v_{2}^{L}\right)}^{q}\right)+\left(1+{\left(\mu_{2}^{U}\right)}^{q}-{\left(v_{2}^{U}\right)}^{q}\right)\right]$ *be the scores of* ${\tilde{p}}_{1}$ *and* ${\tilde{p}}_{2}$, *and let* $H\left({\tilde{p}}_{1}\right)=\frac{{\left(\mu_{1}^{L}\right)}^{q}+{\left(\nu_{1}^{L}\right)}^{q}+{\left(\mu_{1}^{U}\right)}^{q}+{\left(\nu_{1}^{U}\right)}^{q}}{2}$ *and* $H\left({\tilde{p}}_{2}\right)=\frac{{\left(\mu_{2}^{L}\right)}^{q}+{\left(\nu_{2}^{L}\right)}^{q}+{\left(\mu_{2}^{U}\right)}^{q}+{\left(\nu_{2}^{U}\right)}^{q}}{2}$ *be the accuracy degrees of* ${\tilde{p}}_{1}$ *and* ${\tilde{p}}_{2}$, *respectively, then when* $S\left({\tilde{p}}_{1}\right)<S\left({\tilde{p}}_{2}\right)$, ${\tilde{p}}_{1}<{\tilde{p}}_{2}$ *when* $S\left({\tilde{p}}_{1}\right)=S\left({\tilde{p}}_{2}\right)$, *(1) if* $H\left({\tilde{p}}_{1}\right)=H\left({\tilde{p}}_{2}\right)$, *then* ${\tilde{p}}_{1}={\tilde{p}}_{2}$; *(2) if* $H\left({\tilde{p}}_{1}\right)<H\left({\tilde{p}}_{2}\right)$, ${\tilde{p}}_{1}<{\tilde{p}}_{2}$.

**Definition** **5** **[57].** *Let* ${\tilde{p}}_{1}=\left(\left[\mu_{1}^{L},\mu_{1}^{U}\right],\left[\nu_{1}^{L},\nu_{1}^{U}\right]\right)$, ${\tilde{p}}_{2}=\left(\left[\mu_{2}^{L},\mu_{2}^{U}\right],\left[\nu_{2}^{L},\nu_{2}^{U}\right]\right)$ *and* $\tilde{p}=\left(\left[\mu^{L},\mu^{U}\right],\left[\nu^{L},\nu^{U}\right]\right)$ *be three q-RIVOFNs, and some basic rules about them are defined as follows*:

$$\begin{array}{l} (1){\tilde{p}}_{1}⊕{\tilde{p}}_{2}=\left(\left[\begin{array}{l} \sqrt[{q}]{{\left(\mu_{1}^{L}\right)}^{q}+{\left(\mu_{2}^{L}\right)}^{q}-{\left(\mu_{1}^{L}\right)}^{q}{\left(\mu_{2}^{L}\right)}^{q}},\\ \sqrt[{q}]{{\left(\mu_{1}^{U}\right)}^{q}+{\left(\mu_{2}^{U}\right)}^{q}-{\left(\mu_{1}^{U}\right)}^{q}{\left(\mu_{2}^{U}\right)}^{q}} \end{array}\right],\left[v_{1}^{L}v_{2}^{L},v_{1}^{U}v_{2}^{U}\right]\right);\\ (2){\tilde{p}}_{1}⊗{\tilde{p}}_{2}=\left(\left[\mu_{1}^{L}\mu_{2}^{L},\mu_{1}^{U}\mu_{2}^{U}\right],\left[\begin{array}{l} \sqrt[{q}]{{\left(v_{1}^{L}\right)}^{q}+{\left(v_{2}^{L}\right)}^{q}-{\left(v_{1}^{L}\right)}^{q}{\left(v_{2}^{L}\right)}^{q}},\\ \sqrt[{q}]{{\left(v_{1}^{U}\right)}^{q}+{\left(v_{2}^{U}\right)}^{q}-{\left(v_{1}^{U}\right)}^{q}{\left(v_{2}^{U}\right)}^{q}} \end{array}\right]\right);\\ (3)\lambda \tilde{p}=\left(\left[\sqrt[{q}]{1-{\left(1-{\left(\mu_{}^{L}\right)}^{q}\right)}^{\lambda }},\sqrt[{q}]{1-{\left(1-{\left(\mu_{}^{U}\right)}^{q}\right)}^{\lambda }}\right],\left[{\left(v_{}^{L}\right)}^{\lambda },{\left(v_{}^{U}\right)}^{\lambda }\right]\right),\lambda >0;\\ (4){\left(\tilde{p}\right)}^{\lambda }=\left(\left[{\left(\mu_{}^{L}\right)}^{\lambda },{\left(\mu_{}^{U}\right)}^{\lambda }\right],\left[\sqrt[{q}]{1-{\left(1-{\left(v_{}^{L}\right)}^{q}\right)}^{\lambda }},\sqrt[{q}]{1-{\left(1-{\left(v_{}^{U}\right)}^{q}\right)}^{\lambda }}\right]\right),\lambda >0;\\ (5){\tilde{p}}^{c}=\left(\left[v_{}^{L},v_{}^{U}\right],\left[\mu_{}^{L},\mu_{}^{U}\right]\right). \end{array}$$

### 2.2. Some q-RIVOF Aggregation Operators

**Definition** **6** **[57].** *Let* ${\tilde{p}}_{j}=\left(\left[\mu_{j}^{L},\mu_{j}^{U}\right],\left[\nu_{j}^{L},\nu_{j}^{U}\right]\right)\left(j=1,2,\ldots ,n\right)$ *be a list of q-RIVOFNs with weighting vector be* $w_{j}={\left(w_{1},w_{2},\ldots ,w_{n}\right)}^{T}$, *thereby satisfying* $w_{j}\in \left[0,1\right]$ *and* $\sum_{j=1}^{n}w_{j}=1$, *then the q-RIVOFWA operator can be written as*:

(5) $$\begin{array}{l} q-\mathrm{RIVOFWA}\left({\tilde{p}}_{1},{\tilde{p}}_{2},\ldots ,{\tilde{p}}_{n}\right)=\sum_{j=1}^{n}w_{j}{\tilde{p}}_{j}\\ =\left(\left[\sqrt[{q}]{1-\prod_{j=1}^{n}{\left(1-{\left(\mu_{{\tilde{p}}_{j}}^{L}\right)}^{q}\right)}^{w_{j}}},\sqrt[{q}]{1-\prod_{j=1}^{n}{\left(1-\mu {\left(u_{{\tilde{p}}_{j}}^{U}\right)}^{q}\right)}^{w_{j}}}\right],\left[\prod_{j=1}^{n}{\left(v_{{\tilde{p}}_{j}}^{L}\right)}^{w_{j}},\prod_{j=1}^{n}{\left(v_{{\tilde{p}}_{j}}^{U}\right)}^{w_{j}}\right]\right) \end{array}$$

**Definition** **7**  **[57].** *Let* ${\tilde{p}}_{j}=\left(\left[\mu_{j}^{L},\mu_{j}^{U}\right],\left[\nu_{j}^{L},\nu_{j}^{U}\right]\right)\left(j=1,2,\ldots ,n\right)$ *be a list of q-RIVOFNs with weighting vector be* $w_{j}={\left(w_{1},w_{2},\ldots ,w_{n}\right)}^{T}$, *thereby satisfying* $w_{j}\in \left[0,1\right]$ *and* $\sum_{j=1}^{n}w_{j}=1$, *then the q-RIVOFWG operator can be written as*:

(6) $$\begin{array}{l} q-\mathrm{RIVOFWG}\left({\tilde{p}}_{1},{\tilde{p}}_{2},\ldots ,{\tilde{p}}_{n}\right)=\prod_{j=1}^{n}{\left({\tilde{p}}_{j}\right)}^{w_{j}}\\ =\left(\left[\prod_{j=1}^{n}{\left(\mu_{{\tilde{p}}_{j}}^{L}\right)}^{w_{j}},\prod_{j=1}^{n}{\left(\mu_{{\tilde{p}}_{j}}^{U}\right)}^{w_{j}}\right],\left[\sqrt[{q}]{1-\prod_{j=1}^{n}{\left(1-{\left(v_{{\tilde{p}}_{j}}^{L}\right)}^{q}\right)}^{w_{j}}},\sqrt[{q}]{1-\prod_{j=1}^{n}{\left(1-{\left(v_{{\tilde{p}}_{j}}^{U}\right)}^{q}\right)}^{w_{j}}}\right]\right) \end{array}$$

## 3. Traditional VIKOR Model

The VIKOR model, which firstly define by Opricovic and Tzeng [60], is a meaningful tool to investigate MADM problems and has been broadly applied to in the fields of industry, business economy and management in recent years. Assume that there are m alternatives $\left\{A_{1},A_{2},\ldots A_{m}\right\}$, n attributes $\left\{G_{1},G_{2},\ldots G_{n}\right\}$ with weighting vector $\left\{w_{1},w_{2},\ldots w_{n}\right\}$ which meets the condition of $0\leq \;w_{i}\leq 1,\sum_{i=1}^{n}w_{i}=1$ and λ experts with weighting vector $\left\{\omega_{1},\omega_{2},\ldots \omega_{\lambda }\right\}$, respectively, satisfies $0\leq \;\omega_{i}\leq 1,\sum_{i=1}^{t}v_{i}=1$. Set up the matrix $R^{\lambda }={\left[a_{ij}^{\lambda }\right]}_{m\times n},$ i=1,2,…,m,j=1,2,…,n which is used to evaluate each alternative on each indicator, then the traditional VIKOR model can be presented as below.

**Step 1.** Establish the decision matrixes $R^{\lambda }={\left[a_{ij}^{\lambda }\right]}_{m\times n},i=1,2,\ldots ,m,j=1,2,\ldots ,n$ based on expert’s decision making results, and fuse all the evaluation information by using some aggregation operators such as WA operator and WG operator to get fused results matrix $R={\left[a_{ij}^{}\right]}_{m\times n},i=1,2,\ldots ,m,j=1,2,\ldots ,n$;

**Step 2.** Calculate PIS $a_{j}^{+}$ and NIS $a_{j}^{-}$

(7) $$a_{j}^{+}=\left\{\underset{i}{\max}\left(a_{ij}\right)\right\},a_{j}^{-}=\left\{\underset{i}{\min}\left(a_{ij}\right)\right\},i=1,2,\ldots ,m,j=1,2,\ldots ,n$$

**Step 3.** According to the Formula (7) and the attribute weighting vector $w_{j}\left(j=1,2,\ldots ,n\right)$, the results of $\Psi_{i}$ and ${ϒ}_{i}$ which represents the mean and worst group scores of the alternatives $A_{i}$ can be obtained as follows.

(8) $$\Psi_{i}=\sum_{j=1}^{n}w_{j}\frac{d\left(a_{j}^{+},a_{ij}^{}\right)}{d\left(a_{j}^{+},a_{j}^{-}\right)},i=1,2,\ldots ,m,j=1,2,\ldots ,n$$

(9) $${ϒ}_{i}=\underset{j}{\max}\left(w_{j}\frac{d\left(a_{j}^{+},a_{ij}^{}\right)}{d\left(a_{j}^{+},a_{j}^{-}\right)}\right),i=1,2,\ldots ,m,j=1,2,\ldots ,n$$

where $0\leq w_{j}\leq 1$ indicates the weighting vector of attributes which satisfies $\sum_{i=1}^{n}\omega_{i}=1$ and d denotes the q-rung orthopair fuzzy distance measures.

**Step 4.** Calculate the results of $\Theta_{i}$ by following the Equation:

(10) $$\Theta_{i}=\alpha \times \frac{\left(\Psi_{i}-\Psi^{+}\right)}{\left(\Psi^{-}-\Psi^{+}\right)}+\left(1-\alpha \right)\times \frac{\left({ϒ}_{i}-{ϒ}^{+}\right)}{\left({ϒ}^{-}-{ϒ}^{+}\right)}$$

where

(11) $$\Psi^{+}=\underset{i}{\min}\left(\Psi_{i}\right),\Psi^{-}=\underset{i}{\max}\left(\Psi_{i}\right)$$

(12) $${ϒ}^{+}=\underset{i}{\min}\left({ϒ}_{i}\right),{ϒ}^{-}=\underset{i}{\max}\left({ϒ}_{i}\right)$$

where α denotes the coefficient of decision making strategic. α>0.5 means “the maximum group utility”, α=0.5 means equality degree and α<0.5 means the minimum regret degree.

**Step 5.** Then according to $\Theta_{i}$ to select the best alternative, obviously, the smaller the $\Theta_{i}$, the best alternative $A_{i}$ is.

## 4. The VIKOR Model for q-RIVOFNs MAGDM Problems

Assume that there are m alternatives $\left\{A_{1},A_{2},\ldots A_{m}\right\}$, n projects $\left\{G_{1},G_{2},\ldots G_{m}\right\}$ with weighting vector $\left\{w_{1},w_{2},\ldots w_{n}\right\}$ which meets the condition of $0\leq \;w_{i}\leq 1,\sum_{i=1}^{n}w_{i}=1$ and λ experts with weighting vector $\left\{\omega_{1},\omega_{2},\ldots \omega_{\lambda }\right\}$, respectively, the conditions are satisfied $0\leq \;\omega_{i}\leq 1,\sum_{i=1}^{t}v_{i}=1$. Construct the q-RIVOF evaluation matrix $R^{\lambda }={\left[{\tilde{r}}_{ij}^{\lambda }\right]}_{m\times n}={\left(\left[{\left(\mu_{ij}^{\lambda }\right)}^{L},{\left(\mu_{ij}^{\lambda }\right)}^{U}\right],\left[{\left(v_{ij}^{\lambda }\right)}^{L},{\left(v_{ij}^{\lambda }\right)}^{U}\right]\right)}_{m\times n},i=1,2,\ldots ,m,j=1,2,\ldots ,n$, where ${\tilde{r}}_{ij}^{\lambda }=\left(\left[{\left(\mu_{ij}^{\lambda }\right)}^{L},{\left(\mu_{ij}^{\lambda }\right)}^{U}\right],\left[{\left(v_{ij}^{\lambda }\right)}^{L},{\left(v_{ij}^{\lambda }\right)}^{U}\right]\right)$ indicates the q-RIVOF information of the alternative $A_{i}\left(i=1,2,\ldots ,m\right)$ on account of the indicators $G_{j}\left(j=1,2,\ldots ,n\right)$ by expert $D^{\lambda }$. $\left[{\left(\mu_{ij}^{\lambda }\right)}^{L},{\left(\mu_{ij}^{\lambda }\right)}^{U}\right]\in \left[0,1\right]$ denotes the membership degree of alternatives $A_{i}$ satisfies the attribute $G_{j}$ and $\left[{\left(v_{ij}^{\lambda }\right)}^{L},{\left(v_{ij}^{\lambda }\right)}^{U}\right]\in \left[0,1\right]$ is the membership degree of alternatives $A_{i}$ and it indicates that the attribute $G_{j}$ given by the decision maker is not satisfied, respectively, $0\leq {\left({\left(\mu_{ij}^{\lambda }\right)}^{U}\right)}^{q}+{\left({\left(v_{ij}^{\lambda }\right)}^{U}\right)}^{q}\leq 1\left(i=1,2,\ldots ,m,j=1,2,\ldots ,n\right).$ then, based on q-RIVOFSs and traditional VIKOR model, the q-RIVOF-VIKOR model is established to settle MADM problems more reasonably and effectively, the computing steps are simply depicted as follows.

**Step 1.** Give the q-RIVOFNs decision making matrixes $R^{\lambda }={\left[{\tilde{r}}_{ij}^{\lambda }\right]}_{m\times n}={\left(\left[{\left(\mu_{ij}^{\lambda }\right)}^{L},{\left(\mu_{ij}^{\lambda }\right)}^{U}\right],\left[{\left(v_{ij}^{\lambda }\right)}^{L},{\left(v_{ij}^{\lambda }\right)}^{U}\right]\right)}_{m\times n},i=1,2,\ldots ,m,j=1,2,\ldots ,n$ based on expert’s evaluation results, and fuse all the evaluation information by utilizing q-RIVOFWA or q-RIVOFWG operators to obtain the fused matrix $R={\left[{\tilde{r}}_{ij}\right]}_{m\times n}={\left(\left[\mu_{ij}^{L},\mu_{ij}^{U}\right],\left[v_{ij}^{L},v_{ij}^{U}\right]\right)}_{m\times n},$ i=1,2,…,m,j=1,2,…,n;

**Step 2.** Calculate PIS ${\tilde{r}}_{j}^{+}$ and NIS ${\tilde{r}}_{j}^{-}$ by following the Equation:

(13) $${\tilde{r}}_{j}^{+}=\left(\left[{\left(\mu_{ij}^{L}\right)}^{+},{\left(\mu_{ij}^{U}\right)}^{+}\right],\left[{\left(v_{ij}^{L}\right)}^{+},{\left(v_{ij}^{U}\right)}^{+}\right]\right),i=1,2,\ldots ,m,j=1,2,\ldots ,n$$

(14) $${\tilde{r}}_{j}^{-}=\left(\left[{\left(\mu_{ij}^{L}\right)}^{-},{\left(\mu_{ij}^{U}\right)}^{-}\right],\left[{\left(v_{ij}^{L}\right)}^{-},{\left(v_{ij}^{U}\right)}^{-}\right]\right),i=1,2,\ldots ,m,j=1,2,\ldots ,n$$

For benefit attribute:

(15) $${\tilde{r}}_{j}^{+}=\left(\left[\underset{i}{\max}\left(\mu_{ij}^{L}\right),\underset{i}{\max}\left(\mu_{ij}^{U}\right)\right],\left[\underset{i}{\min}\left(v_{ij}^{L}\right),\underset{i}{\min}\left(v_{ij}^{U}\right)\right]\right),i=1,2,\ldots ,m,j=1,2,\ldots ,n$$

(16) $${\tilde{r}}_{j}^{-}=\left(\left[\underset{i}{\min}\left(\mu_{ij}^{L}\right),\underset{i}{\min}\left(\mu_{ij}^{U}\right)\right],\left[\underset{i}{\max}\left(v_{ij}^{L}\right),\underset{i}{\max}\left(v_{ij}^{U}\right)\right]\right),i=1,2,\ldots ,m,j=1,2,\ldots ,n$$

For cost attribute:

(17) $${\tilde{r}}_{j}^{+}=\left(\left[\underset{i}{\min}\left(\mu_{ij}^{L}\right),\underset{i}{\min}\left(\mu_{ij}^{U}\right)\right],\left[\underset{i}{\max}\left(v_{ij}^{L}\right),\underset{i}{\max}\left(v_{ij}^{U}\right)\right]\right),i=1,2,\ldots ,m,j=1,2,\ldots ,n$$

(18) $${\tilde{r}}_{j}^{-}=\left(\left[\underset{i}{\max}\left(\mu_{ij}^{L}\right),\underset{i}{\max}\left(\mu_{ij}^{U}\right)\right],\left[\underset{i}{\min}\left(v_{ij}^{L}\right),\underset{i}{\min}\left(v_{ij}^{U}\right)\right]\right),i=1,2,\ldots ,m,j=1,2,\ldots ,n$$

**Step 3.** On the basis of the Equations (17) and (18) and $w_{j}\left(j=1,2,\ldots ,n\right)$, the results of $\Psi_{i}$ and ${ϒ}_{i}$ which represents the mean and worst group scores of the alternatives $A_{i}$ can be obtained as follows.

(19) $$\Psi_{i}=\sum_{j=1}^{n}w_{j}\frac{d\left({\tilde{r}}_{j}^{+},{\tilde{r}}_{ij}^{}\right)}{d\left({\tilde{r}}_{j}^{+},{\tilde{r}}_{j}^{-}\right)},i=1,2,\ldots ,m,j=1,2,\ldots ,n$$

(20) $${ϒ}_{i}=\underset{j}{\max}\left(w_{j}\frac{d\left({\tilde{r}}_{j}^{+},{\tilde{r}}_{ij}^{}\right)}{d\left({\tilde{r}}_{j}^{+},{\tilde{r}}_{j}^{-}\right)}\right),i=1,2,\ldots ,m,j=1,2,\ldots ,n$$

where $0\leq w_{j}\leq 1$ indicates the weighting vector of attributes which satisfies $\sum_{i=1}^{n}\omega_{i}=1$ and d denotes the q-rung orthopair fuzzy distance measures. For the traditional normalized Hamming distance (HD) measures or Euclidean distance measures (ED) are limited to deal with some special situations, thus, we shall use the combination form of three distance measures mentioned as follows.

(21) $$d\left({\tilde{r}}_{ij},{\tilde{r}}_{tj}\right)=\sum_{k=1}^{3}\chi_{k}d^{k}\left({\tilde{r}}_{ij},{\tilde{r}}_{tj}\right),\chi_{k}\in \left[0,1\right],\sum_{k=1}^{3}\chi_{k}=1.$$

where $0\leq \chi_{k}\leq 1$ indicates the weighting vector of distance measures $d^{k}$ and

(22) $$d^{1}\left({\tilde{r}}_{ij},{\tilde{r}}_{tj}\right)=\frac{\left(\left|{\left(\mu_{ij}^{L}\right)}^{q}-{\left(\mu_{tj}^{L}\right)}^{q}\right|+\left|{\left(\mu_{ij}^{U}\right)}^{q}-{\left(\mu_{tj}^{U}\right)}^{q}\right|+\left|{\left(v_{ij}^{L}\right)}^{q}-{\left(v_{tj}^{L}\right)}^{q}\right|+\left|{\left(v_{ij}^{U}\right)}^{q}-{\left(v_{tj}^{U}\right)}^{q}\right|\right)}{4}$$

(23) $$d^{2}\left({\tilde{r}}_{ij},{\tilde{r}}_{tj}\right)=\frac{\left|\left\{{\left(\mu_{ij}^{L}\right)}^{q}+{\left(\mu_{ij}^{U}\right)}^{q}-{\left(v_{ij}^{L}\right)}^{q}-{\left(v_{ij}^{U}\right)}^{q}\right\}-\left\{{\left(\mu_{tj}^{L}\right)}^{q}+{\left(\mu_{tj}^{U}\right)}^{q}-{\left(v_{tj}^{L}\right)}^{q}-{\left(v_{tj}^{U}\right)}^{q}\right\}\right|}{4}$$

(24) $$d^{3}\left({\tilde{r}}_{ij},{\tilde{r}}_{tj}\right)=\left\{\begin{array}{c} \max\left\{\begin{array}{cc} \; & \frac{2-{\left(\mu_{ij}^{L}\right)}^{q}-{\left(\mu_{ij}^{U}\right)}^{q}-{\left(v_{ij}^{L}\right)}^{q}-{\left(v_{ij}^{U}\right)}^{q}}{4},\\ \; & \frac{2-{\left(\mu_{tj}^{L}\right)}^{q}-{\left(\mu_{tj}^{U}\right)}^{q}-{\left(v_{tj}^{L}\right)}^{q}-{\left(v_{tj}^{U}\right)}^{q}}{4} \end{array}\right\}\\ -\min\left\{\begin{array}{c} \frac{2-{\left(\mu_{ij}^{L}\right)}^{q}-{\left(\mu_{ij}^{U}\right)}^{q}-{\left(v_{ij}^{L}\right)}^{q}-{\left(v_{ij}^{U}\right)}^{q}}{4},\\ \frac{2-{\left(\mu_{tj}^{L}\right)}^{q}-{\left(\mu_{tj}^{U}\right)}^{q}-{\left(v_{tj}^{L}\right)}^{q}-{\left(v_{tj}^{U}\right)}^{q}}{4} \end{array}\right\} \end{array}\right\}$$

**Step 4.** Calculate the results of $\Theta_{i}$ by following the Equation:

(25) $$\Theta_{i}=\alpha \times \frac{\left(\Psi_{i}-\Psi^{+}\right)}{\left(\Psi^{-}-\Psi^{+}\right)}+\left(1-\alpha \right)\times \frac{\left({ϒ}_{i}-{ϒ}^{+}\right)}{\left({ϒ}^{-}-{ϒ}^{+}\right)}$$

where

(26) $$\Psi^{+}=\underset{i}{\min}\left(\Psi_{i}\right),\Psi^{-}=\underset{i}{\max}\left(\Psi_{i}\right)$$

(27) $${ϒ}^{+}=\underset{i}{\min}\left({ϒ}_{i}\right),{ϒ}^{-}=\underset{i}{\max}\left({ϒ}_{i}\right)$$

where α denotes the coefficient of decision making strategic. α>0.5 means “the maximum group utility”, α=0.5 means equality degree and α<0.5 means the minimum regret degree.

**Step 5.** According to $\Theta_{i}$ to select the best alternative, obviously, the smaller the $\Theta_{i}$, the best alternative $A_{i}$ is.

## 5. The Numerical Example

### 5.1. Numerical for q-RIVOFNs MAGDM Problems

The supplier terms of an enterprise is undoubtedly very important, and in the future will be an even more important influence on the quality of a vendor’s business, as it will affect the business of purchasing, production, inventory and sales, and so on. The relationship between suppliers and future enterprise is not a simple relationship between management and managed, suppliers will become a strategic partner companies, it is a win-win relationship. So supplier preliminary evaluation and selection is quite important. Medical supplies products have their own characteristics to distinguish it from other types of products, which can be distinguished from their production, transportation, marketing, and other aspects. It can be seen that supplier selection of medical consumption products is the classical MADM or MAGDM issue [61,62,63,64,65,66,67,68,69,70,71,72]. In this subsection, an example for supplier selection of medical consumption products with q-RIVOF information shall be presented in order to demonstrate the method proposed in this paper. There is a panel with five possible medical consumption products suppliers. $\eta_{i}\left(i=1,2,3,4,5\right)$ to sort. Experts select four attributes to appraise the five feasible construction projects: ① G_1_ is the environmental improvement quality; ② G_2_ is the transportation convenience of suppliers; ③ G_3_ is the green image; ④ G_4_ is the environmental competencies. The five feasible medical consumption products suppliers $\eta_{i}\left(i=1,2,3,4,5\right)$ are to be evaluated using the q q-RIVOF information under the above four attributes by three experts $D^{\lambda }$ (Assume the weighting vector of experts is $\left(0.35,0.25,0.40\right)$ and attribute index’s weighting vector is $\left(0.27,0.37,0.16,0.20\right)$).

Next, we make use of the VIKOR technique with q-RIVOFNs developed for medical consumption products supplier selection.

**Step 1.** Give the q-RIVOFNs decision making matrixes $R^{\lambda }={\left[{\tilde{r}}_{ij}^{\lambda }\right]}_{m\times n}={\left(\left[{\left(\mu_{ij}^{\lambda }\right)}^{L},{\left(\mu_{ij}^{\lambda }\right)}^{U}\right],\left[{\left(v_{ij}^{\lambda }\right)}^{L},{\left(v_{ij}^{\lambda }\right)}^{U}\right]\right)}_{m\times n},i=1,2,\ldots ,m,j=1,2,\ldots ,n$ as follows.

Then according to q-RIVOFWA operator and q-RIVOFNs given in Table 1, Table 2 and Table 3, the fused results matrix can be obtained as follows (Suppose q=4).

**Step 2.** Calculate PIS ${\tilde{r}}_{j}^{+}$ and NIS ${\tilde{r}}_{j}^{-}$ by Equations (13) and (14), for all attributes are benefit we can easily gain the results of (PIS) $\eta^{+}$ and (NIS) $\eta^{-}$ as follows;

$$\eta^{+}=\left\{\begin{array}{l} \left(\left[0.6709,0.8095],[0.1275,0.2305\right]\right),\left(\left[0.6243,0.7462],[0.2430,0.2430\right]\right),\\ \left(\left[0.6245,0.7335],[0.7335,0.3824\right]\right),\left(\left[0.6312,0.7766],[0.1803,0.4302\right]\right) \end{array}\right\}$$

$$\eta^{-}=\left\{\begin{array}{l} \left(\left[0.3975,0.5017],[0.6051,0.7445\right]\right),\left(\left[0.3411,0.4487],[0.4800,0.6448\right]\right),\\ \left(\left[0.3108,0.5282],[0.4278,0.5975\right]\right),\left(\left[0.4630,0.5576],[0.3903,0.5537\right]\right) \end{array}\right\}$$

**Step 3.** According to the Formulas (19) and (20) and $w_{j}\left(j=1,2,\ldots ,n\right)$, the results of $\Psi_{i}$ and ${ϒ}_{i}$ which denote the mean and the worst group scores of alternative $\eta_{i}$ can be obtained. Suppose the weights of distance measures $d^{k}$ are (0.3, 0.4, 0.3), then the results of combination distance can be calculated in Table 4.

Then the results of $\Psi_{i}$ and ${ϒ}_{i}$ are calculated as:

$$\Psi_{1}=0.6112,\Psi_{2}=0.0292,\Psi_{3}=0.8467,\Psi_{4}=0.4445,\Psi_{5}=0.5890,$$

$${ϒ}_{1}=0.5890,\;{ϒ}_{2}=0.0166,\;{ϒ}_{3}=0.3088,\;{ϒ}_{4}=0.1818,\;{ϒ}_{5}=0.2919.$$

**Step 4.** On the basis of $\Psi_{i}$ and ${ϒ}_{i}$ obtained by above steps, we can calculate the results of $\Theta_{i}$, the results are recorded as below. (Let α=0.4)

$$\Theta_{1}=0.7172,\Theta_{2}=0.0000,\Theta_{3}=1.0000,\Theta_{4}=0.5424,\Theta_{5}=0.8392.$$

**Step 5.** According to $\Theta_{i}$ to select the best alternative, obviously, the smaller value the $\Theta_{i}$ is, the best alternative $\eta_{i}$ is. Apparently, the ordering of $\eta_{i}$ is $\Theta_{2}>\Theta_{4}>\Theta_{1}>\Theta_{5}>\Theta_{3}$, and the best choice is $\eta_{2}$.

### 5.2. Comparative Analyses for q-RIVOFNs MAGDM Problems

In this subsection, we shall compare our presented VIKOR model for q-RIVOFNs with other existing q-RIVOF decision making tools including q-RIVOFWA operator and q-RIVOFWG operator proposed by Wang, Gao, Wei and Wei [57] to explain the model we developed is scientifically valid. Using the fused q-RIVOFNs results of Table 5 and the weights of attributes, the fused results depicted by q-RIVOFNs of each alternative are listed in Table 6.

According to the score function of q-RIVOFNs, the score results $s\left(\eta_{i}\right)$ of each alternative can be determined as follows.

For q-RIVOFWA operator:

$$s\left(\eta_{1}\right)=0.5461,s\left(\eta_{2}\right)=0.6228,s\left(\eta_{3}\right)=0.5092,s\left(\eta_{4}\right)=0.5599,s\left(\eta_{5}\right)=0.5489.$$

For q-RIVOFWG operator:

$$s\left(\eta_{1}\right)=0.5380,s\left(\eta_{2}\right)=0.6188,s\left(\eta_{3}\right)=0.4785,s\left(\eta_{4}\right)=0.5462,s\left(\eta_{5}\right)=0.5267.$$

Then the ordering of alternatives by q-RIVOFWA and q-RIVOFWG operators is listed in Table 7.

Compare the results of the q-RIVOF-VIKOR model with q-RIVOFWA and q-ROIVFWG operators, the aggregation results are a little bit different in ranking of alternatives but the optimal scheme is the same. However, q-RIVOF-VIKOR model has the remarkable characteristics of considering the compromise between group utility maximization and individual regret minimization and can be more accuracy and valid in MAGDM problems.

## 6. Conclusions

In this manuscript, the q-RIVOF-VIKOR model based on the traditional VIKOR model is presented. Firstly, we started with a review of the concept of q-RIVOFSs and introduced the score function, accuracy function, operation rules, and some aggregation operators of q-RIVOFNs. Furthermore, we combined the traditional VIKOR technique with q-RIVOFNs information, the q-RIVOF-VIKOR model is built and the calculational steps are detailedly given. The proposed model considers the compromise between group utility maximization and individual regret minimization, which is proved to be more accurate and effective. Finally, the new model is illustrated by taking the supplier selection of medical consumer products as an example, and the advantages of the new method are further illustrated by comparing q-RIVOF-VIKOR model with two q-RIVOFNs aggregation operators. In the future, the q-RIVOF-VIKOR model can be used in many other uncertain and fuzzy environments, such as risk analysis [73,74,75,76,77,78,79,80,81,82,83,84].

## Figures and Tables

**Table 1:** The q-RIVOFNs information given by $D^{1}$.

	G_1_	G_2_	G_3_	G_4_
$\eta_{1}$	([0.7,0.8],[0.4,0.5])	([0.2,0.4],[0.5,0.8])	([0.5,0.6],[0.3,0.4])	([0.6,0.7],[0.6,0.8])
$\eta_{2}$	([0.8,0.9],[0.2,0.3])	([0.5,0.6],[0.1,0.2])	([0.6,0.7],[0.5,0.6])	([0.6,0.8],[0.3,0.4])
$\eta_{3}$	([0.5,0.6],[0.7,0.8])	([0.3,0.4],[0.4,0.5])	([0.6,0.8],[0.5,0.7])	([0.4,0.5],[0.2,0.3])
$\eta_{4}$	([0.6,0.7],[0.3,0.4])	([0.2,0.5],[0.5,0.6])	([0.5,0.7],[0.3,0.4])	([0.6,0.8],[0.2,0.4])
$\eta_{5}$	([0.5,0.9],[0.3,0.6])	([0.4,0.6],[0.7,0.8])	([0.3,0.4],[0.5,0.6])	([0.6,0.7],[0.2,0.3])

**Table 2:** The q-RIVOFNs information given by $D^{2}$.

	G_1_	G_2_	G_3_	G_4_
$\eta_{1}$	([0.6,0.7],[0.2,0.3])	([0.1,0.4],[0.6,0.8])	([0.3,0.7],[0.2,0.5])	([0.4,0.6],[0.5,0.7])
$\eta_{2}$	([0.5,0.6],[0.1,0.2])	([0.8,0.9],[0.5,0.6])	([0.4,0.6],[0.2,0.3])	([0.7,0.8],[0.4,0.5])
$\eta_{3}$	([0.3,0.4],[0.5,0.6])	([0.2,0.4],[0.5,0.7])	([0.5,0.6],[0.2,0.3])	([0.6,0.7],[0.5,0.6])
$\eta_{4}$	([0.3,0.5],[0.5,0.7])	([0.4,0.5],[0.7,0.8])	([0.4,0.5],[0.2,0.5])	([0.5,0.9],[0.3,0.4])
$\eta_{5}$	([0.4,0.8],[0.2,0.4])	([0.2,0.3],[0.6,0.7])	([0.4,0.7],[0.2,0.3])	([0.3,0.6],[0.4,0.8])

**Table 3:** The q-RIVOFNs information given by $D^{3}$.

	G_1_	G_2_	G_3_	G_4_
$\eta_{1}$	([0.5,0.6],[0.3,0.4])	([0.6,0.7],[0.1,0.2])	([0.2,0.4],[0.5,0.8])	([0.3,0.5],[0.2,0.3])
$\eta_{2}$	([0.5,0.7],[0.1,0.2])	([0.4,0.6],[0.4,0.5])	([0.7,0.8],[0.2,0.3])	([0.6,0.7],[0.4,0.5])
$\eta_{3}$	([0.2,0.4],[0.6,0.8])	([0.4,0.5],[0.1,0.2])	([0.4,0.7],[0.6,0.8])	([0.3,0.4],[0.6,0.9])
$\eta_{4}$	([0.4,0.5],[0.2,0.3])	([0.7,0.8],[0.3,0.6])	([0.6,0.8],[0.1,0.5])	([0.1,0.2],[0.6,0.7])
$\eta_{5}$	([0.3,0.6],[0.4,0.5])	([0.5,0.6],[0.3,0.4])	([0.1,0.3],[0.4,0.5])	([0.5,0.8],[0.1,0.4])

**Table 4:** The results of combination distance.

	G_1_	G_2_	G_3_	G_4_
$d\left(\eta_{1},\eta^{+}\right)$	0.0585	0.0750	0.0829	0.0860
$d\left(\eta_{2},\eta^{+}\right)$	0.0062	0.0004	0.0012	0.0086
$d\left(\eta_{3},\eta^{+}\right)$	0.1797	0.1019	0.0435	0.1031
$d\left(\eta_{4},\eta^{+}\right)$	0.1142	0.0600	0.0235	0.0281
$d\left(\eta_{5},\eta^{+}\right)$	0.0497	0.0964	0.0929	0.0398
$d\left(\eta^{-},\eta^{+}\right)$	0.1797	0.1221	0.1024	0.1031

**Table 5:** The fused q-RIVOFNs matrix.

	G_1_	G_2_
$\eta_{1}$	([0.6171,0.7179],[0.2998,0.4025])	([0.4834,0.5860],[0.2750,0.4595])
$\eta_{2}$	([0.6709,0.7975],[0.1275,0.2305])	([0.6243,0.7462],[0.2604,0.3798])
$\eta_{3}$	([0.3975,0.5017],[0.6051,0.7445])	([0.3411,0.4487],[0.2430,0.3770])
$\eta_{4}$	([0.4932,0.5993],[0.2899,0.4101])	([0.5763,0.6855],[0.4434,0.6448])
$\eta_{5}$	([0.4224,0.8095],[0.3042,0.5041])	([0.4316,0.5634],[0.4800,0.5864])
	**G_3_**	**G_4_**
$\eta_{1}$	([0.3975,0.5884],[0.3326,0.5581])	([0.4879,0.6171],[0.3695,0.5227])
$\eta_{2}$	([0.6245,0.7335],[0.2757,0.3824])	([0.6312,0.7675],[0.3617,0.4625])
$\eta_{3}$	([0.5187,0.7277],[0.4278,0.5975])	([0.4630,0.5576],[0.3903,0.5537])
$\eta_{4}$	([0.5337,0.7249],[0.1747,0.4625])	([0.5005,0.7766],[0.3435,0.5004])
$\eta_{5}$	([0.3108,0.5282],[0.3637,0.4691])	([0.5209,0.7335],[0.1803,0.4302])

**Table 6:** The fused results of each alternative $\eta_{i}$

q-RIVOFWA Operator	q-RIVOFWG Operator
$\begin{array}{l} \eta_{1}=\left(\left[0.5246,0.6383],[0.3078,0.4693\right]\right)\\ \eta_{2}=\left(\left[0.6395,0.7642],[0.2314,0.3456\right]\right)\\ \eta_{3}=\left(\left[0.4254,0.5631],[0.3741,0.5267\right]\right)\\ \eta_{4}=\left(\left[0.5361,0.6984],[0.3237,0.5143\right]\right)\\ \eta_{5}=\left([0.4411,0.6986],[0.3337,0.5106]\right) \end{array}$	$\begin{array}{l} \eta_{1}=\left(\left[0.5013,0.6258],[0.3162,0.4829\right]\right)\\ \eta_{2}=\left(\left[0.6380,0.7619],[0.2799,0.3813\right]\right)\\ \eta_{3}=\left(\left[0.4041,0.5218],[0.4706,0.6092\right]\right)\\ \eta_{4}=\left(\left[0.5307,0.6839],[0.3726,0.5533\right]\right)\\ \eta_{5}=\left(\left[0.4227,0.6482],[0.3985,0.5258\right]\right) \end{array}$

**Table 7:** Order of alternatives by q-RIVOFWA and q-RIVOFWG operators.

	Order
q-RIVOFWA	$\eta_{2}>\eta_{4}>\eta_{5}>\eta_{1}>\eta_{3}$
q-RIVOFWG	$\eta_{2}>\eta_{4}>\eta_{1}>\eta_{5}>\eta_{3}$
q-RIVOF-VIKOR	$\eta_{2}>\eta_{4}>\eta_{1}>\eta_{5}>\eta_{3}$

## References

[1] Cali S., Balaman S.Y. (2019). A novel outranking based multi criteria group decision making methodology integrating ELECTRE and VIKOR under intuitionistic fuzzy environment. Expert Syst. Appl..

[2] Feng J.B., Li M., Li Y.S. (2018). Study of Decision Framework of Shopping Mall Photovoltaic Plan Selection Based on DEMATEL and ELECTRE III with Symmetry under Neutrosophic Set Environment. Symmetry.

[3] Ji P., Zhang H.Y., Wang J.Q. (2018). Selecting an outsourcing provider based on the combined MABAC-ELECTRE method using single-valued neutrosophic linguistic sets. Comput. Ind. Eng..

[4] Zandi A., Roghanian E. (2013). Extension of Fuzzy ELECTRE based on VIKOR method. Comput. Ind. Eng..

[5] Pamucar D., Petrovic I., Cirovic G. (2018). Modification of the Best-Worst and MABAC methods: A novel approach based on interval-valued fuzzy-rough numbers. Expert Syst. Appl..

[6] Peng X., Yang Y. (2016). Pythagorean Fuzzy Choquet Integral Based MABAC Method for Multiple Attribute Group Decision Making. Int. J. Intell. Syst..

[7] Yu S.-M., Wang J., Wang J.-Q. (2017). An Interval Type-2 Fuzzy Likelihood-Based MABAC Approach and Its Application in Selecting Hotels on a Tourism Website. Int. J. Fuzzy Syst..

[8] Bekar E.T., Cakmakci M., Kahraman C. (2016). Fuzzy COPRAS method for performance measurement in total productive maintenance: A comparative analysis. J. Bus. Econ. Manag..

[9] Wang Z.L., You J.X., Liu H.C., Wu S.M. (2017). Failure Mode and Effect Analysis using Soft Set Theory and COPRAS Method. Int. J. Comput. Intell. Syst..

[10] Wei G.W. (2010). Extension of TOPSIS method for 2-tuple linguistic multiple attribute group decision making with incomplete weight information. Knowl. Inf. Syst..

[11] Wei G.W., Lin R., Zhao X.F., Wang H.J. (2010). TOPSIS-Based Linear-Programming Methodology for Multiple Attribute Decision Making with Incomplete Weight Information in Intuitionistic Fuzzy Setting. Inf.-Int. Interdiscip. J..

[12] Zhang N., Wei G.W. (2013). Extension of VIKOR method for decision making problem based on hesitant fuzzy set. Appl. Math. Model..

[13] Wang J., Wei G.W., Lu M. (2018). TODIM Method for Multiple Attribute Group Decision Making under 2-Tuple Linguistic Neutrosophic Environment. Symmetry.

[14] Huang Y.H., Wei G.W. (2018). TODIM method for Pythagorean 2-tuple linguistic multiple attribute decision making. J. Intell. Fuzzy Syst..

[15] Xu D.S., Wei C., Wei G.W. (2017). TODIM Method for Single-Valued Neutrosophic Multiple Attribute Decision Making. Information.

[16] Li X.Y., Wei G.W. (2014). GRA method for multiple criteria group decision making with incomplete weight information under hesitant fuzzy setting. J. Intell. Fuzzy Syst..

[17] Wei G.W. (2010). GRA method for multiple attribute decision making with incomplete weight information in intuitionistic fuzzy setting. Knowl.-Based Syst..

[18] Wei G.W., Zhao X.F., Wang H.J., Lin R. (2010). GRA Model for Selecting an ERP System in Trapezoidal intuitionistic Fuzzy Setting. Inf.-Int. Interdiscip. J..

[19] Du Y., Liu P.D. (2011). Extended fuzzy VIKOR method with intuitionistic trapezoidal fuzzy numbers. Inf.-Int. Interdiscip. J..

[20] Park J.H., Cho H.J., Kwun Y.C. (2011). Extension of the VIKOR method for group decision making with interval-valued intuitionistic fuzzy information. Fuzzy Optim. Decis. Mak..

[21] Qin J.D., Liu X.W., Pedrycz W. (2015). An extended VIKOR method based on prospect theory for multiple attribute decision making under interval type-2 fuzzy environment. Knowl.-Based Syst..

[22] Ghadikolaei A.S., Madhoushi M., Divsalar M. (2018). Extension of the VIKOR method for group decision making with extended hesitant fuzzy linguistic information. Neural Comput. Appl..

[23] Wang J., Wei G.W., Lu M. (2018). An Extended VIKOR Method for Multiple Criteria Group Decision Making with Triangular Fuzzy Neutrosophic Numbers. Symmetry.

[24] Narayanamoorthy S., Geetha S., Rakkiyappan R., Joo Y.H. (2019). Interval-valued intuitionistic hesitant fuzzy entropy based VIKOR method for industrial robots selection. Expert Syst. Appl..

[25] Yang W., Pang Y.F., Shi J.R., Wang C.J. (2018). Linguistic hesitant intuitionistic fuzzy decision-making method based on VIKOR. Neural Comput. Appl..

[26] Wang L., Zhang H.Y., Wang J.Q., Li L. (2018). Picture fuzzy normalized projection-based VIKOR method for the risk evaluation of construction project. Appl. Soft Comput..

[27] Wu Z.B., Xu J.P., Jiang X.L., Zhong L. (2019). Two MAGDM models based on hesitant fuzzy linguistic term sets with possibility distributions: VIKOR and TOPSIS. Inf. Sci..

[28] Zadeh L.A. (1965). Fuzzy Sets. Inf. Control.

[29] Atanassov K. (1986). Intuitionistic fuzzy sets. Fuzzy Sets Syst..

[30] Atanassov K. (1989). More on intuitionistic fuzzy sets. Fuzzy Sets Syst..

[31] Yager R.R. Pythagorean Fuzzy Subsets. Proceedings of the 2013 Joint IFSA World Congress and NAFIPS Annual Meeting (IFSA/NAFIPS).

[32] Yager R.R. (2014). Pythagorean membership grades in multicriteria decision making. IEEE Trans. Fuzzy Syst..

[33] Zhang X.L., Xu Z.S. (2014). Extension of TOPSIS to Multiple Criteria Decision Making with Pythagorean Fuzzy Sets. Int. J. Intell. Syst..

[34] Peng X.D., Yang Y. (2015). Some Results for Pythagorean Fuzzy Sets. Int. J. Intell. Syst..

[35] Reformat M.Z., Yager R.R., Laurent A., Strauss O., BouchonMeunier B., Yager R.R. (2014). Suggesting Recommendations Using Pythagorean Fuzzy Sets illustrated Using Netflix Movie Data. Information Processing and Management of Uncertainty in Knowledge-Based Systems Pt I.

[36] Gou X.J., Xu Z.S., Ren P.J. (2016). The Properties of Continuous Pythagorean Fuzzy Information. Int. J. Intell. Syst..

[37] Garg H. (2016). A New Generalized Pythagorean Fuzzy Information Aggregation Using Einstein Operations and Its Application to Decision Making. Int. J. Intell. Syst..

[38] Wu S.J., Wei G.W. (2017). Pythagorean fuzzy Hamacher aggregation operators and their application to multiple attribute decision making. Int. J. Knowl.-Based Intell. Eng. Syst..

[39] Zeng S.Z., Chen J.P., Li X.S. (2016). A Hybrid Method for Pythagorean Fuzzy Multiple-Criteria Decision Making. Int. J. Inf. Technol. Decis. Mak..

[40] Ren P.J., Xu Z.S., Gou X.J. (2016). Pythagorean fuzzy TODIM approach to multi-criteria decision making. Appl. Soft Comput..

[41] Wei G.W., Lu M. (2018). Pythagorean Fuzzy Maclaurin Symmetric Mean Operators in Multiple Attribute Decision Making. Int. J. Intell. Syst..

[42] Maclaurin C. (1729). A second letter to Martin Folkes, Esq.; concerning the roots of equations, with demonstration of other rules of algebra. Philos. Trans..

[43] Wei G.W. (2017). Pythagorean fuzzy interaction aggregation operators and their application to multiple attribute decision making. J. Intell. Fuzzy Syst..

[44] Wei G.W., Lu M. (2018). Pythagorean fuzzy power aggregation operators in multiple attribute decision making. Int. J. Intell. Syst..

[45] Wei G.W., Wei Y. (2018). Similarity measures of Pythagorean fuzzy sets based on the cosine function and their applications. Int. J. Intell. Syst..

[46] Liang D.C., Zhang Y.R.J., Xu Z.S., Darko A.P. (2018). Pythagorean fuzzy Bonferroni mean aggregation operator and its accelerative calculating algorithm with the multithreading. Int. J. Intell. Syst..

[47] Liang D.C., Xu Z.S., Darko A.P. (2017). Projection Model for Fusing the Information of Pythagorean Fuzzy Multicriteria Group Decision Making Based on Geometric Bonferroni Mean. Int. J. Intell. Syst..

[48] Zhu B., Xu Z.S., Xia M.M. (2012). Dual Hesitant Fuzzy Sets. J. Appl. Math..

[49] Wang H.J., Zhao X.F., Wei G.W. (2014). Dual hesitant fuzzy aggregation operators in multiple attribute decision making. J. Intell. Fuzzy Syst..

[50] Wei G.W., Lu M. (2017). Dual hesitant pythagorean fuzzy Hamacher aggregation operators in multiple attribute decision making. Arch. Control Sci..

[51] Peng X.D., Yuan H.Y., Yang Y. (2017). Pythagorean Fuzzy Information Measures and Their Applications. Int. J. Intell. Syst..

[52] Yager R.R. (2017). Generalized Orthopair Fuzzy Sets. Ieee Trans. Fuzzy Syst..

[53] Liu P.D., Wang P. (2018). Some q-Rung Orthopair Fuzzy Aggregation Operators and their Applications to Multiple-Attribute Decision Making. Int. J. Intell. Syst..

[54] Wei G.W., Wei C., Wang J., Gao H., Wei Y. (2019). Some q-rung orthopair fuzzy maclaurin symmetric mean operators and their applications to potential evaluation of emerging technology commercialization. Int. J. Intell. Syst..

[55] Wei G.W., Gao H., Wei Y. (2018). Some q-rung orthopair fuzzy Heronian mean operators in multiple attribute decision making. Int. J. Intell. Syst..

[56] Yang W., Pang Y.F. (2019). New q-rung orthopair fuzzy partitioned Bonferroni mean operators and their application in multiple attribute decision making. Int. J. Intell. Syst..

[57] Wang J., Gao H., Wei G.W., Wei Y. (2019). Methods for Multiple-Attribute Group Decision Making with q-Rung Interval-Valued Orthopair Fuzzy Information and Their Applications to the Selection of Green Suppliers. Symmetry.

[58] Liu P.D., Liu W.Q. (2019). Multiple-attribute group decision-making based on power Bonferroni operators of linguistic q-rung orthopair fuzzy numbers. Int. J. Intell. Syst..

[59] Xu Y., Shang X., Wang J., Wu W., Huang H. (2018). Some q-Rung Dual Hesitant Fuzzy Heronian Mean Operators with Their Application to Multiple Attribute Group Decision-Making. Symmetry.

[60] Opricovic S., Tzeng G.H. (2007). Extended VIKOR method in comparison with outranking methods. Eur. J. Oper. Res..

[61] Lu J.P., Tang X.Y., Wei G.W., Wei C., Wei Y. (2019). Bidirectional project method for dual hesitant Pythagorean fuzzy multiple attribute decision-making and their application to performance assessment of new rural construction. Int. J. Intell. Syst..

[62] Lu J.P., Wei C., Wu J., Wei G.W. (2019). TOPSIS Method for Probabilistic Linguistic MAGDM with Entropy Weight and Its Application to Supplier Selection of New Agricultural Machinery Products. Entropy.

[63] Wei G.W., Wang J., Lu M., Wu J., Wei C. (2019). Similarity Measures of Spherical Fuzzy Sets Based on Cosine Function and Their Applications. IEEE Access.

[64] Wei G.W., Wang J., Wei C., Wei Y., Zhang Y. (2019). Dual Hesitant Pythagorean Fuzzy Hamy Mean Operators in Multiple Attribute Decision Making. IEEE Access.

[65] Zhang S.Q., Wei G.W., Gao H., Wei C., Wei Y. (2019). EDAS method for multiple criteria group decision making with picture fuzzy information and its application to green suppliers selections. Technol. Econ. Dev. Econ..

[66] Deng X.M., Gao H. (2019). TODIM method for multiple attribute decision making with 2-tuple linguistic Pythagorean fuzzy information. J. Intell. Fuzzy Syst..

[67] Gao H., Lu M., Wei Y. (2019). Dual hesitant bipolar fuzzy hamacher aggregation operators and their applications to multiple attribute decision making. J. Intell. Fuzzy Syst..

[68] Li Z.X., Lu M. (2019). Some novel similarity and distance and measures of Pythagorean fuzzy sets and their applications. J. Intell. Fuzzy Syst..

[69] Lu J.P., Wei C. (2019). TODIM method for Performance Appraisal on Social-Integration-based Rural Reconstruction with Interval-Valued Intuitionistic Fuzzy Information. J. Intell. Fuzzy Syst..

[70] Wei G., Wei C., Wu J., Wang H. (2019). Supplier Selection of Medical Consumption Products with a Probabilistic Linguistic MABAC Method. Int. J. Environ. Res. Public Health.

[71] He T., Wei G., Lu J., Wei C., Lin R. (2019). Pythagorean 2-Tuple Linguistic Taxonomy Method for Supplier Selection in Medical Instrument Industries. Int. J. Environ. Res. Public Health.

[72] He T.T., Wei G.W., Lu J.P., Wei C., Lin R. (2019). Pythagorean 2-Tuple Linguistic VIKOR Method for Evaluating Human Factors in Construction Project Management. Mathematics.

[73] Wang J., Gao H., Lu M. (2019). Approaches to strategic supplier selection under interval neutrosophic environment. J. Intell. Fuzzy Syst..

[74] Wang R. (2019). Research on the Application of the Financial Investment Risk Appraisal Models with Some Interval Number Muirhead Mean Operators. J. Intell. Fuzzy Syst..

[75] Wu L.P., Gao H., Wei C. (2019). VIKOR method for financing risk assessment of rural tourism projects under interval-valued intuitionistic fuzzy environment. J. Intell. Fuzzy Syst..

[76] Wu L.P., Wang J., Gao H. (2019). Models for competiveness evaluation of tourist destination with some interval-valued intuitionistic fuzzy Hamy mean operators. J. Intell. Fuzzy Syst..

[77] Wei G.W., Wu J., Wei C., Wang J., Lu J.P. (2019). Models for MADM With 2-Tuple Linguistic Neutrosophic Dombi Bonferroni Mean Operators. IEEE Access.

[78] Wei G.W., Zhang S.Q., Lu J.P., Wu J., Wei C. (2019). An Extended Bidirectional Projection Method for Picture Fuzzy MAGDM and Its Application to Safety Assessment of Construction Project. IEEE Access.

[79] Bozanic D., Tešić D., Milićević J. (2018). A hybrid fuzzy AHP-MABAC model: Application in the Serbian Army—The selection of the location for deep wading as a technique of crossing the river by tanks. Decis. Mak..

[80] Fazlollahtabar H., Smailbašić A., Stević Ž. (2019). FUCOM method in group decision-making: Selection of forklift in a warehouse. Decis. Mak..

[81] Karabašević D., Popović G., Stanujkić D., Maksimović M., Sava C. (2019). An approach for hotel type selection based on the single-valued intuitionistic fuzzy numbers. Int. Rev..

[82] Pamučar D., Božanić D. (2019). Selection of a location for the development of multimodal logistics center: Application of single-valued neutrosophic MABAC model. Oper. Res. Eng. Sci..

[83] Naeini A.B., Mosayebi A., Mohajerani N. (2019). A hybrid model of competitive advantage based on Bourdieu capital theory and competitive intelligence using fuzzy Delphi and ism-gray Dematel (study of Iranian food industry). Int. Rev..

[84] Hassanpour M., Pamucar D. (2019). Evaluation of Iranian household appliance industries using MCDM models. Oper. Res. Eng. Sci..

